# COVID-19: systemic pathology and its implications for therapy

**DOI:** 10.7150/ijbs.65911

**Published:** 2022-01-01

**Authors:** Qi Shen, Jie Li, Zhan Zhang, Shuang Guo, Qiuhong Wang, Xiaorui An, Haocai Chang

**Affiliations:** 1 MOE Key Laboratory of Laser Life Science & Institute of Laser Life Science, College of Biophotonics, South China Normal University, Guangzhou 510631, China.; 2 Guangdong Provincial Key Laboratory of Laser Life Science, College of Biophotonics, South China Normal University, Guangzhou 510631, China.; 3 Department of Neurology, Sun Yat-sen Memorial Hospital, Sun Yat-sen University, Guangzhou 510120, China.; 4 Guangdong Province Key Laboratory of Brain Function and Disease, Zhongshan School of Medicine, Sun Yat-sen University, Guangzhou 510120, China.; 5 Dermatology Hospital, Southern Medical University, Guangzhou 510091, China.; 6 Qilu Cell Therapy Technology Co., Ltd, Jinan 250000, China.

**Keywords:** SARS-CoV-2, immune system, nervous system, reproductive system, motor system, immunotherapy

## Abstract

Responding to the coronavirus disease 2019 (COVID-19) pandemic has been an unexpected and unprecedented global challenge for humanity in this century. During this crisis, specialists from the laboratories and frontline clinical personnel have made great efforts to prevent and treat COVID-19 by revealing the molecular biological characteristics and epidemic characteristics of the severe acute respiratory syndrome coronavirus 2 (SARS-CoV-2). Currently, SARS-CoV-2 has severe consequences for public health, including human respiratory system, immune system, blood circulation system, nervous system, motor system, urinary system, reproductive system and digestive system. In the review, we summarize the physiological and pathological damage of SARS-CoV-2 to these systems and its molecular mechanisms followed by clinical manifestation. Concurrently, the prevention and treatment strategies of COVID-19 will be discussed in preclinical and clinical studies. With constantly unfolding and expanding scientific understanding about COVID-19, the updated information can help applied researchers understand the disease to build potential antiviral drugs or vaccines, and formulate creative therapeutic ideas for combating COVID-19 at speed.

## 1. Introduction

Coronavirus disease 2019 (COVID-19) with acute respiratory disease and potentially severe pneumonia has resulted in unimaginable consequence to public health and loss of human life due to severe acute respiratory syndrome coronavirus 2 (SARS-CoV-2). As the end of 5 November 2021, there have been 248,467,363 confirmed cases of COVID-19, including 5,027,183 deaths (https://covid19.who.int/). The focus of global efforts is to develop safe and effective vaccines against COVID-19, and some vaccines are provisionally licensed for COVID-19 prevention. However, the population of COVID-19 patients is still surging in the case of public vaccination.

**Figure 1:**
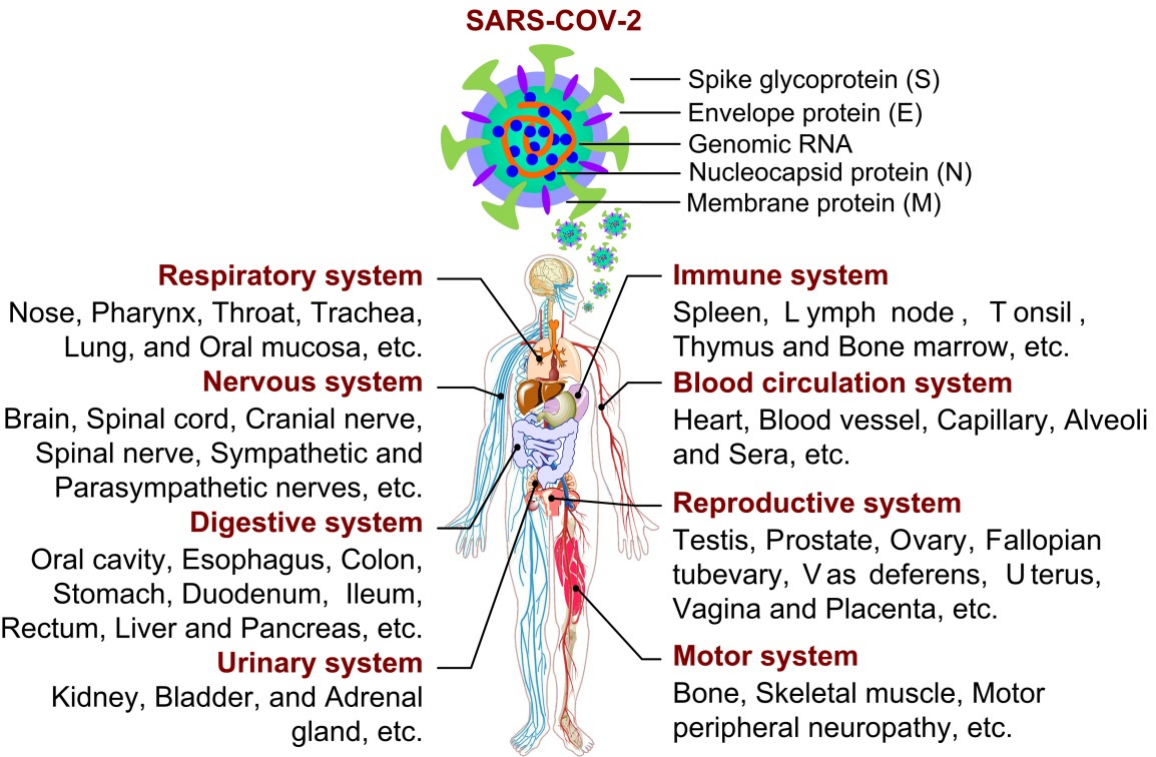
Structure of SARS-CoV-2 and its infection on tissues and organs in eight major systems of human organism.

As reported, SARS-CoV-2 is a single-stranded positive-sense RNA virus, whose genome size varies from 29.8 ~ 29.9 kb 1, and whose genome encodes 4 structural proteins (spike surface glycoprotein (S), membrane protein, envelope protein and nucleocapsid protein), 16 non-structural proteins (NSP1-16) and some accessory proteins (ORF3a, ORF3b, ORF6, ORF7a, ORF7b, ORF8, ORF9b and ORF10) 2-4 (Fig. 1). With unremitting efforts of scientific researchers around the world, the structure and function of these proteins have been more understood. However, effective treatment including drug or vaccine is still not available for COVID-19, the study on SARS-CoV-2 still needs to be explored. So far, few studies have systematically investigated the physiological and pathological features of SARS-CoV-2 from a multisystem perspective (Fig. 1). In this review, the information about the characteristics, damage and treatment of SARS-CoV-2 is summarized and updated by our limited understanding. Importantly, we have tried to give a full insight into physiological effects and action mechanisms of SARS-CoV-2 in order to find potential therapeutic strategies or control measures in this way to combat the ongoing infection.

## 2. Affect and Effect of COVID-19

### 2.1. COVID-19 on respiratory system

Obviously, respiratory system is the first to bear the brunt of SARS-CoV-2, which as a danger signaling is sent to immune system. As a receptor of S surface protein on SARS-CoV-2, angiotensin converting enzyme II (ACE2) is mainly expressed in type-2 alveolar (AT2) cells (83% in average) 5 and other certain cells such as epithelial cells, which exists widely in various tissues and organs including oral mucosa 6, airway 7, lung 8, colon 9, kidney 10 and prostate 11. Depending on the context, lungs are directly under the strongest attack to cause lung inflammation and functional injury. As a result, pulmonary fibrosis occurs frequently in the infected patients, along with dry cough, dyspnea and fatigue symptoms.

Pulmonary fibrosis is characterized by the excessive deposition of extracellular matrix components including collagen and fibronectin, mainly originating from the persistence of fibroblast proliferation and activation. A molecular explanation for pulmonary fibrosis is that fibroblast growth factor (FGF) and transforming growth factor (TGF), as inducers of collagen and fibronectin 12, 13, are both high levels in COVID-19 patients 14, 15. As reported, other upregulated pro-inflammatory cytokines, such as platelet-derived growth factor (PDGF), vascular endothelial growth factor (VEGF), tumor necrosis factor α (TNFα) and interleukin-6 (IL-6), also involved in the process 16. Another important aspect is that ongoing AT2 epithelial cell injury can recruit fibroblasts to fibrotic loci, and then fibroblasts differentiate into myofibroblasts to produce extracellular matrix proteins 16, 17.

### 2.2. COVID-19 on immune system

An effective immune response against SARS-CoV-2 requires two arms of the immune system, the innate immune system and the adaptive immune system. The innate immune system, as the first line of defense of the immune system, is responsible for rapidly recognizing the infection and triggering alarms, in which a range of innate immune cells are involved. On the other hand, the adaptive immune system is essential for controlling and clearing the viral infection, the three fundamental components of which (CD4^+^ T cells, CD8^+^ T cells, B cells) cooperate with each other to regulate the antigen-specific immune responses. Therefore, understanding the interaction between SARS-CoV-2 and the immune system is of great importance for pathogenesis, disease progression, treatment strategies, and vaccine design of COVID-19.

#### 2.2.1. Innate immune response

The innate immune response correlates with the severity of COVID-19, which has been proved by a range of studies 18, 19. The innate immune system against SARS-CoV-2 infection has three main pathways: (1) limiting virus replication in infected cells; (2) development of antiviral state in the infection site, including recruitment of various innate immune cells; (3) initiating the adaptive immunity 20. All of these pathways require the involvement of multiple types of innate immune cells, of which the more common cells are granulocytes, monocytes, macrophages and natural killer (NK) cells, but there are also many other immune cells including different dendritic cells (DCs), innate lymphoid cells, and mast cells.

##### 2.2.1.1. Granulocytes and monocytes

**Figure 2:**
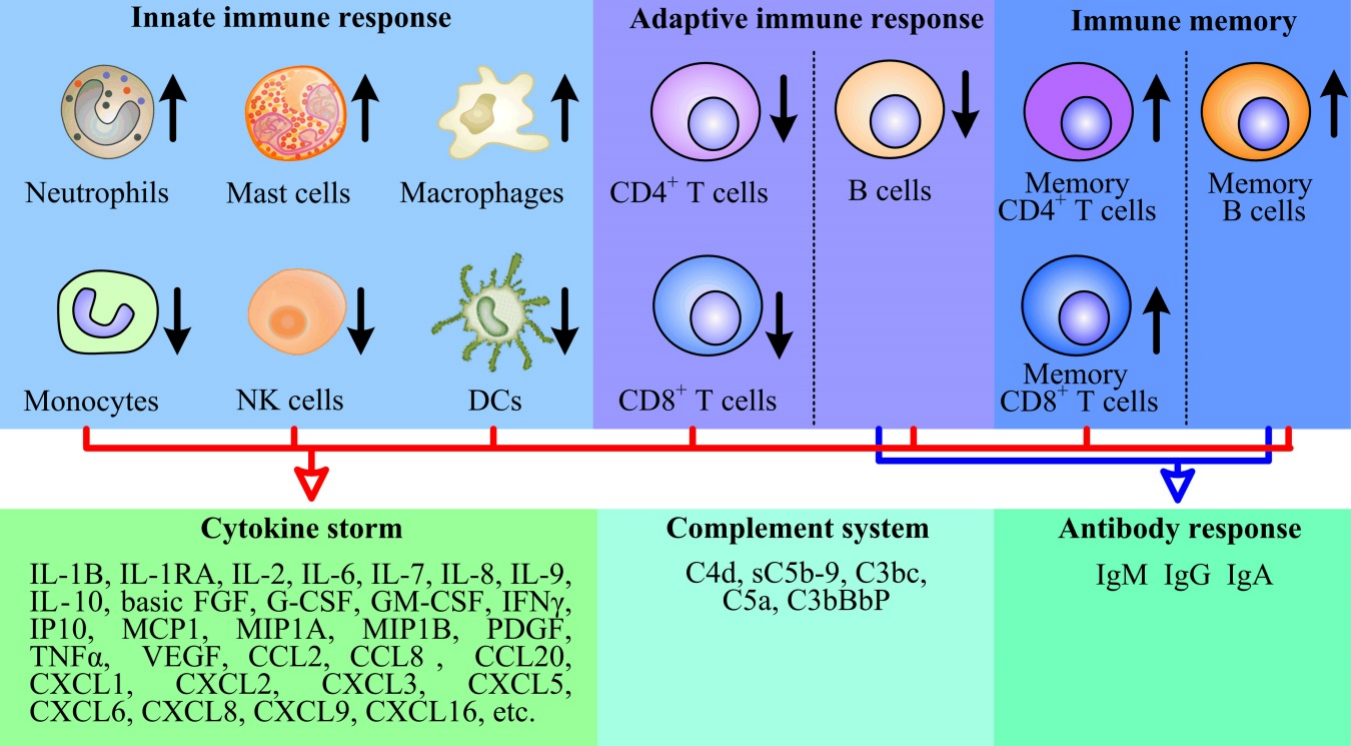
The immunopathology of SARS-CoV-2. SARS-CoV-2 reduced the number of monocytes, NK cells, DCs, CD4^+^ T cells, CD8^+^ T cells and B cells. Conversely, the virus increased the number of neutrophils, mast cells, macrophages, memory CD4^+^ T cells, memory CD8^+^ T cells and memory B cells to some extent, and triggered complement system responses, then the host produced a strong and harmful cytokine storm, and a weak and favorable antibody response.

A few studies have revealed that increases in monocytes, neutrophils and eosinophils correlate with the severity of disease in COVID-19 patients 21 (Fig. 2). Liu found that the neutrophil-to-lymphocyte ratio (NLR) could be served as a risk factor for early-stage prediction of COVID-19 patients, whose age is over 50 years old and NLR is more than 3.13 are more likely predicted to develop critical illness 22. A prominent higher proportion of neutrophils and activated mast cells was also observed in the bronchoalveolar lavage fluid (BALF) of COVID-19 patients 18 (Fig. 2). The fact that chemoattractants for neutrophils were upregulated during COVID-19 19, 23, 24 suggested that granulocytes may significantly contribute to pathogenesis.

CD16^+^ monocytes were depleted in COVID-19 patients and were remodeled with a phenotypic shift toward CD14^+^ monocytes 24. Inflammatory HLA-DR^hi^ CD11c^hi^ CD14^+^ monocytes can be considered as a hallmark of mild COVID-19 patients 25. Moreover, decrease of CD14^lo^ CD16^hi^ monocytes and the appearance of dysfunctional monocytes with low expression of human leukocyte antigen class DR (HLA-DR) and high expression of alarming S100 were related to the severe COVID-19 patients 24, 25. Interestingly, Guo *et al*. found a unique subpopulation of monocytes, with high expression of inflammatory genes, specifically present in the severe COVID-19 cases. These inflammatory genes were potentially regulated by the transcription factors ATF3, NFIL3, and HIVEP2 26. And COVID-19 patients were reported to have greater abundance of CD14^+^ IL-1β^+^ and IFN-activated monocytes compared to healthy controls 27. These data suggest the potential risk of inflammatory cytokine storms caused by monocytes. In addition, ISGs upregulation in monocytes was heterogeneous, with no clear relevance of COVID-19 severity 24.

##### 2.2.1.2. Macrophages and DCs

Proinflammatory monocyte-derived macrophages had higher abundance in the BALF from severe COVID-19 than mild cases 28. Further analysis of the heterogeneity of macrophages in severe and mild patients found that the mild patients highly expressed FABP4, while the severe patients highly expressed FCN1 and SPP1. These data suggest the imbalance of lung macrophage populations in COVID-19 patients. In general, a highly proinflammatory macrophage microenvironment is present in the lungs of severe COVID-19 patients, which may contribute to recruitment of other innate immune cells and tissue damage. Additional data showed that macrophages in the lower airways had a stronger inflammatory signature than those within the upper airways 29. Conventional DCs (cDCs) and plasmacytoid DCs (pDCs) have been reported to significantly decrease in BALFs of patients with severe COVID-19 28. Sánchez-Cerrillo *et al*. found that CD1c^+^ cDCs preferentially migrated from blood to lungs in severe COVID-19 cases, whereas CD141^+^ cDCs and CD123^hi^ pDCs were depleted from blood and were also absent in the lungs 30. Another study found a decrease in the resting DCs and an increase in the activated DCs in the lungs of COVID-19 cases 24.

##### 2.2.1.3. NK cells

Accumulating studies have reported low levels of NK cells in the peripheral blood of COVID-19 patients with moderate and severe disease 24, 31, 32. However, two reports assessing the immune cells in BALF of COVID-19 patients have revealed that NK cells are increased at this infection site 28, 29. Maucourant *et al*. found low numbers but a strong activation phenotype (Ki-67^+^, HLA-DR^+^, CD69^+^) of NK cells in peripheral blood of COVID-19 patients and adaptive NK cells, with high expression of perforin, NKG2C, and Ksp37, was increased in circulation of patients with severe COVID-19 33. Analysis of NK cell transcriptomic signatures furthermore confirmed that the increased expression of cytotoxic marker PRF1 and repair marker DDIT4 in NK cells was associated with recovered COVID-19 patients 34. In contrast, another study found NKG2A, an inhibitory receptor, has been upregulated on peripheral NK cells, meanwhile, the expressions of the activation markers CD107a, IFN-γ, IL-2, and TNFα were decreased. These results suggest the functional exhaustion of peripheral NK cells in COVID-19 patients 31. Moreover, in convalescent patients, the frequency of NK cells was restored 31, 35.

##### 2.2.1.4. Complement system

The complement system is a key component of innate immune response to clear pathogens and serves as a danger-sensing alarming system. Patients with COVID-19 have been reported with the activation of the complement system, and intense complement activation was related to severe disease 36. COVID-19 patients showed the complement activation in their lung, sera, and other organ tissue 37, 38. Mannose-associated serine protease 2 (MASP-2) is the critical component for mediating activation of the lectin pathway. Nucleocapsid protein of SARS-CoV-2 was found to interact with MASP-2 38, which led to a strong complement activation and further aggravated lung injury. Complement activation products in COVID-19 patients included the classical/lectin (C4d), alternative (C3bBbP) and common pathway (C3bc, C5a, and sC5b-9), the lectin pathway recognition molecule mannose-binding lectin (MBL). Among which, sC5b-9 and C4d were significantly higher in patients with respiratory failure than without respiratory failure 37 (Fig. 2). Similarly, complement 6 and complement factor B, as the components of complement system, have been reported to be elevated in serum of patients with COVID-19, suggesting that the complement activation is an early immune response mechanism 39.

##### 2.2.1.5. Cytokine storm

It was observed that the cytokine storm was associated with disease severity of COVID-19. A clinical investigation of 41 confirmed cases infected with SARS-CoV-2 in China revealed that the initial plasma concentrations of IL-1B, IL-1RA, IL-7, IL-8, IL-9, IL-10, basic FGF, G-CSF, GM-CSF, IFN-γ, IP10, MCP1, MIP1A, MIP1B, PDGF, TNFα, and VEGF were higher compared to healthy individuals 21. Further analysis of both ICU and non-ICU patients found that ICU patients exhibited higher plasma concentrations of IL-2, IL-7, IL-10, G-CSF, IP10, MCP1, MIP1A, and TNFα than non-ICU patients 21. Similarly, additional studies quantified cytokines and chemokines in serum samples of COVID-19 patients, and the results showed remarkable increases in circulating levels of IL-6, IL-1RA, CCL2, CCL8 CXCL2, CXCL8, CXCL9, and CXCL16 19. The significant increased chemokines are likely responsible for the recruitment of neutrophil (CXCL1, CXCL2, CXCL6) and monocyte (CCL2 and CCL8) to the lungs 19. However, it is worth mentioning that whole blood RNA levels of cytokines were not always consistent with protein plasma levels. For example, IL-6 had not detected an increase at the transcriptional level in the peripheral blood of COVID-19 patients, but there is a large amount of IL-6 protein. TNFα was only moderately upregulated at the transcriptional level, while circulating TNFα was significantly increased 40, 41.

The interferon system plays an important role in antiviral immunity, and it is critical to understand the IFN response in COVID-19. An immune analysis based on a cohort of 50 COVID-19 patients revealed that type I IFN response was high in mild to moderate patients, whereas severe patients had significantly lower type I IFN responses than mild-to-moderate patients 41. In contrast to severe influenza, COVID-19 patients exhibited a hyperinflammatory profile in their peripheral blood mononuclear cells, with a particular upregulation of TNF/IL-1β-driven inflammatory responses, and type I IFN responses coexist with TNF/IL-1β-driven inflammation in monocytes from severe COVID-19 patients 42. These observations were consistent with an enhanced expression of ISGs in patients with COVID-19 41, 43. Zhou *et al*. reported that the expression of 83 ISGs was significantly upregulated in COVID-19 patients, including IFITMs with direct antiviral activity, compared with community-acquired pneumonia patients 18. However, in *in vitro* infection model using cell lines, SARS-CoV-2 only induced low levels of type I and type II IFNs, as well as moderate levels of ISGs and a distinct proinflammatory cytokine profiles including IL-1B, IL-6 and TNF, and many chemokines (CCL20, CXCL1, CXCL2, CXCL3, CXCL5, CXCL6 and CXCL16) 19, 40, resulting in efficiently restricting SARS-CoV-2 infection. A longitudinal immunological analysis of COVID-19 revealed that IFNα and IFNλ in moderate patients were high levels during the early phase and then declined, whereas IFNα and IFNλ in severe patients showed a continuous increase in overall 23. The dynamic changes of type I IFNs in COVID-19 patients suggest that type I IFNs do not control the replication of SARS-CoV-2 *in vivo* but are important drivers of the disease progression.

#### 2.2.2. Adaptive immune response

The adaptive immune response is critical for controlling and eliminating most viral infections. Studies of COVID-19 patients have observed that adaptive immune response limits COVID-19 disease severity and balanced CD4^+^ T cell, CD8^+^ T cell, and antibody responses are protective, significantly associated with milder disease. Understanding the quantity and function changes in the three branches (B cells, CD4^+^ T cells and CD8^+^ T cells) of adaptive immune system in COVID-19 patients will provide insights into immunity and pathogenesis of SARS-CoV-2 infection, and the same knowledge also contributes to the vaccine development and evaluation of candidate vaccines.

##### 2.2.2.1. Lymphopenia

Lymphopenia is prevalent in patients with COVID-19 and is correlated with increased disease severity. Patients who suffered COVID-19 showed lower total blood lymphocyte compared to healthy individuals, including a significant decrease in counts of CD4^+^ T cells, CD8^+^ T cells, NK cells and NKT cells 23. The lymphocyte percentages were mostly lower than 20% in severe patients, who were more likely to exhibit lymphopenia than mild/moderate patients 44. Moreover, further analysis revealed that patients with severe COVID-19 had a remarkable decrease in T cells counts, but not B cells, especially CD8^+^ T cells compared with moderate patients. These clinical data suggest that lymphopenia can be used as one of the effective indicators of disease severity and prognostic evaluation in COVID-19 patients.

##### 2.2.2.2. CD4^+^ T cells

Studies have noted that circulating SARS-CoV-2-specific CD8^+^ and CD4^+^ T cells existed in 70% and 100% of convalescent COVID-19 patients respectively 45, indicating that almost all SARS-CoV-2 infections elicit the T cell response, especially CD4^+^ T cell responses. Structural proteins (spike, membrane, and nucleocapsid) of SARS-CoV-2 are the prominent targets of SARS-CoV-2-specific CD4^+^ T cells. Additional CD4^+^ T cell responses also against NSP3, NSP4, open reading frame (ORF) 3a, and ORF8 45. SARS-CoV-2-specific CD4^+^ T cells can be detected as early as 2-4 days after the onset of symptoms 46, 47, which occurred in mild COVID-19 patients, accelerating viral clearance. Conversely, the delayed appearance of SARS-CoV-2-specific CD4^+^ T cells was associated with severe or fatal COVID-19 (> 22 days after the onset of symptoms in some cases) 46, 47. Moreover, SARS-CoV-2-specific CD4^+^ T cells in severe COVID-19 patients displayed low antigen avidity and clonality 48.

Virus-specific CD4^+^ T cells have the capacity for differentiation into multiple helper and effector cell types in response to SARS-CoV-2, which recruit innate cells and provide help to B cells and CD8^+^ T cells, with the abilities of direct antiviral activities and facilitating tissue repair. Studies have been revealed that the SARS-CoV-2-specific CD4^+^ T cells from both acute and convalescent COVID-19 patients mainly produced IFN-γ, TNF and IL-2, the classical cytokines signature during type I T helper (Th1) cell responses, with direct antiviral functions 45, 46, 49. Another branch of CD4^+^ T cells in immune responses against SARS-CoV-2 is the differentiated T follicular helper cells (Tfh), whose primary function is to assist B cells in proliferation and neutralizing antibody production, participating in humoral immunity. It has been found that SARS-CoV-2-specific circulating Tfh cells (cTfh) were a substantial fraction of the SARS-CoV-2-specific CD4^+^ T cells in acute and convalescent COVID-19 patients 46. And, the higher cell frequencies of SARS-CoV-2-specific cTfh have been associated with lower disease severity 46. Cytotoxic T helper cells (CD4-CTLs) are related cells with direct cytotoxic activity. Besides cytotoxicity-associated transcripts, the SARS-CoV-2-specific CD4-CTL were highly enriched for transcripts encoding for a range of chemokines such as CCL3, CCL4 and XCL1. These chemokines participate in the recruitment of myeloid cells (neutrophils, monocytes, and macrophages), NK cells, and DCs to the sites of viral infection 50. According to the relevant data, the proportion of CD4-CTLs and antigen-specific regulatory T cells (Tregs) exhibited an obvious negative correlation 51.

##### 2.2.2.3. CD8^+^ T cells

CD8^+^ T cells are important in clearing viral infections due to their capacity for killing infected cells. In COVID-19 patients, the presence of SARS-CoV-2-specific CD8^+^ T cells has been related to less severe disease 46. Similarly to SARS-CoV-2-specific CD4^+^ T cells, SARS-CoV-2 CD8^+^ T cells are specific for multiple SARS-CoV-2 antigens, such as spike, nucleocapsid, membrane and ORF3a protein 45. SARS-CoV-2-specific CD8^+^ T cells can be detected as early as day 1 post-symptom onset 52. The data showed that SARS-CoV-2-specific CD8^+^ T cells were detected in 87% of convalescent COVID-19 cases but only in 53% of acute cases 46. In the acute COVID-19 cases, SARS-CoV-2-specific CD8^+^ T cells were characterized by activation marker (CD38, HLA-DR, and Ki-67) and predominantly expressed IFN-γ, granzyme B, perforin, and CD107a, with cytotoxic effector functions 45, 53. However, SARS-CoV-2-specific CD8^+^ T cells in convalescent COVID-19 patients tended to an early differentiated memory phenotype (CCR7^+^ CD127^+^ CD45RA^-/+^ TCF1^+^) 54. From the foregoing, increasing the number of CD4 and CD8 T cells through the combination of other therapeutic approaches may be a better method for the treatment of COVID-19 patients, including the use of drug (such as CD3 antibody and CD28 antibody 55) and drug-free (such as phototherapy 56) therapeutic strategies.

##### 2.2.2.4. Antibody response

The SARS-CoV-2 infected individuals exhibited seroconversion within 20 days after symptom onset 24, 57, 58. Seroconversion for immunoglobulin (Ig) M and IgG antibodies against SARS-CoV-2 occurred simultaneously or sequentially 57. A study based on 173 COVID-19 patients revealed that the seroconversion rates for total antibodies, IgM, and IgG were 93.1%, 82.7%, and 64.7%, respectively 59. The spike and nucleocapsid of SARS-CoV-2 are primary antigens testing for seroconversion 60. Spike is the main target of neutralizing antibodies against SARS-CoV-2, and its receptor binding domain (RBD) is the target of more than 90% of neutralizing antibodies in COVID-19 patients 60, 61, whereas some other neutralizing antibodies instead target the NTD 61. Moreover, most COVID-19 patients had detectable IgG and IgA responses to spike and nucleocapsid, whereas IgM responses were limited to spike and undetectable for nucleocapsid 60.

Among most SARS-CoV-2-infected patients, neutralizing antibodies developed rapidly. Zhao's data showed that the antibodies were less than 40% within 1 week since onset, and could rapidly increase to 100.0% (total antibodies), 94.3% (IgM), and 79.8% (IgG) within 15 days 59. And neutralizing antibodies titers stayed relatively stable for at least 5 months 62. Additionally, it is worth noting that SARS-CoV-2 neutralizing antibody titers are positively correlated with COVID-19 disease severity 57, 60, 63. Consistently, most convalescent cases who recover from COVID-19 do not exhibit high levels of neutralizing antibodies activity. These antibodies had little somatic hypermutation and were highly enriched for the usage of gene VH1-69, VH3-30-3, and VH1-24 61. Furthermore, the production of SARS-CoV-2 neutralizing antibodies did not require affinity maturation. Collectively, these data suggest that the neutralizing antibodies against SARS-CoV-2 are relatively easy to generate.

##### 2.2.2.5. Immune memory

Immunological memory is composed of memory CD4^+^ T cells, memory CD8^+^ T cells and memory B cells. All three major types of immune memory are substantially generated after SARS-CoV-2 infection. About 95% of individuals kept immune memory at ~6 months after SARS-CoV-2 infection 64.

About 90% of subjects are seropositive for SARS-CoV-2 neutralizing antibodies at 6 to 8 months after symptom onset 64. SARS-CoV-2 RBD IgM and IgG titers steadily decreased within 1 to 6 months after infection, whereas IgA was less declined relatively 65. One cross-sectional analysis of COVID-19 subjects has revealed that SARS-CoV-2 specific memory B cells were detectable in all subjects at 6 months post-infection. Moreover, the frequencies of memory B cells specific to spike, RBD, and nucleocapsid increased over time in the following 4 to 5 months post-infection and then plateaued 64. RBD memory B cells had undergone affinity maturation after infection 6 months and expressed the increased potency neutralizing antibodies, indicating continuous evolution of humoral immunity 65.

The memory CD4^+^ T cells predominantly consists of Th1 and Tfh cells 64, 66. The majority of SARS-CoV-2 memory CD8^+^ T cells were mainly terminally differentiated effector memory cells (T_EMRA_), with small populations of central memory (T_CM_) and effector memory (T_EM_) 64. A few studies have assessed memory CD4^+^ T cells and CD8^+^ T cells at least 6 months after infection. One study found that ~90% of subjects were positive for SARS-CoV-2 memory CD4^+^ T cells and ~50% were positive for memory CD8^+^ T cells after 6 months primary infection 64. There was a steady decline in SARS-CoV-2 memory CD8^+^ T cells and CD4^+^ T cells within 8 months after infection.

### 2.3. COVID-19 on blood circulation system

#### 2.3.1. Hematologic Parameters of COVID -19

Patients with COVID-19 usually present with abnormal hematologic changes, including white blood cells reduction, lymphopenia, and thrombocytopenia. Based on the clinical data of 1099 COVID-19 cases provided by Guan *et al*. 67, the vast majority of patients on admission presented with lymphocytopenia (83.2%), whereas 36.2% had thrombocytopenia, and 33.7% showed leukopenia. And these hematological abnormalities were more prominent in severe versus non-severe cases.

Several factors may contribute to COVID-19 associated lymphopenia. On the one hand, previous study showed that lymphocytes expressed the ACE2 receptor on their surface 6, thus SARS-CoV-2 directly infected those cells and eventually led to cell lysis. On the other hand, the virus particles disseminate through the respiratory mucosa and infect other cells, inducing a cytokine storm in the body. It is characterized by markedly increased levels of interleukins (IL-2, IL-6, IL-8), G-CSF, IFN-γ inducible protein (IP-10) and TNFα, which promote lymphocyte apoptosis 68-70. Furthermore, massive cytokine activation may be also associated with atrophy of lymphoid organs, including the spleen, and further impairs lymphocyte turnover 71.

#### 2.3.2. COVID-19 and cardiovascular system

Patients with prior cardiovascular disease are at higher risk for adverse events from COVID-19. Individuals without a history of cardiovascular disease are at risk for incident cardiovascular complications 72. The vast majority of COVID-19 patients with previous cardiovascular disease presented with cardiovascular complications including hypertension (40%) 73, 74, whereas 10% had coronary heart disease, 17% had cardiac arrhythmias, and 4% showed heart failure 22, 75. Patients with more severe clinical presentations present with comorbidities such as hypertension (58%), heart disease (25%), and arrhythmias (44%) 76, 77. Additionally, cardiovascular manifestations, during COVID-19, are mostly represented by acute cardiac injury (ACI), defined as a rise of cardiac troponin values, with or without ejection fraction decline or electrocardiographic abnormalities. The prevalence of acute cardiac injury among COVID-19 patients was 10-23% 21.

Furthermore, COVID-19 patients are also at an increased risk of venous thromboembolism and their main coagulation parameters (elevated D-Dimer levels, fibrin degradation products) are also altered, especially in patients with severe manifestations 72. The direct effects of COVID-19 or the indirect effects of infection, such as severe illness and hypoxia, may predispose patients to thrombotic events. Preliminary reports suggest that hemostatic abnormalities, including disseminated intravascular coagulation (DIC), occur in patients affected by SARS-CoV-2 78, 79. Additionally, the severe inflammatory response, critical illness, and underlying traditional risk factors may all predispose to thrombotic events 79, 80. Overall, patients with cardiovascular disease represent more than 20% of all fatal cases, with a case fatality rate of 10.5% 75.

### 2.4. COVID-19 on nervous system

**Figure 3:**
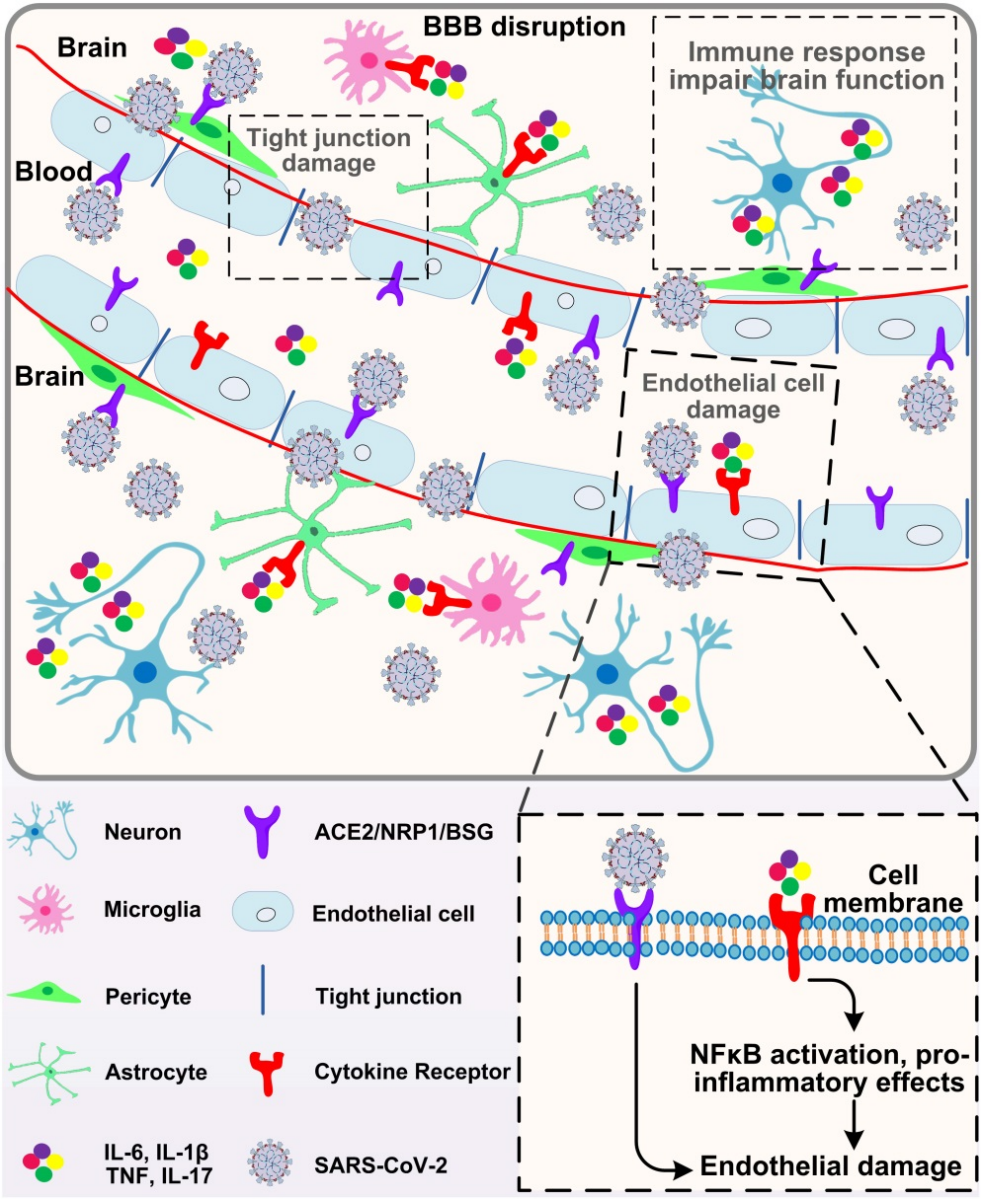
Brain entry of SARS-CoV-2. Circulating SARS-CoV-2 and cytokines act on endothelial cells, leading to inflammation and BBB opening. Once in the perivascular space, these factors induce inflammation in vascular parietal cells, microglia and macrophages resident in the brain. The cytokines may affect the function of neurons and lead to cytokine sickness, which is a potential cause of COVID-19 encephalopathy.

Similar to other coronaviruses (mainly SARS-CoV-1, MERS-CoV and OC43), the possibility of SARS-CoV-2 invading the central nervous system has been proposed 81. *In vivo* studies in human ACE2 transgenic mice have shown that SARS-CoV-2 can infect neurons and cause neuronal death in an ACE2 dependent manner 82. In brain cells derived from human pluripotent stem cells, dopaminergic neurons (rather than cortical neurons or microglia) are particularly sensitive to SARS-CoV-2 infection 83. However, the results of clinicopathological studies on the detection of virus in brain or cerebrospinal fluid (CSF) are different. Some studies have shown that SARS-CoV-2 RNA exists in the brain or cerebrospinal fluid of patients with encephalopathy or encephalitis after death, but the level is very low 84. Other studies have failed to detect viral invasion, even if there is evidence of CSF inflammation 85, 86. Recent studies have shown that SARS-CoV-2 can enter the brain through damaged blood-brain barrier (BBB), olfactory bulb/olfactory nerve or lymphatic vessels 87. SARS-CoV-2 can infect and directly cause endothelial cell damage, increase BBB permeability, and cause edema formation. It has been reported that ACE2 is expressed in physiological conditions in neurons and glial cells, making the brain more vulnerable to SARS-CoV-2 infection 88. Infected neurons release inflammatory molecules and activate nearby immune cells, including mast cells, endothelial cells, pericytes, neurons, microglia and astrocytes. When ACE2 is expressed in brain endothelial cells, SARS-CoV-2 infection can cause cerebral hemorrhage and BBB dysfunction 89, leading to endothelial cell damage, brain edema formation, neuronal death and cognitive decline (Fig. 3). In addition, researches demonstrated that ACE2 is also expressed in pericytes, another apart of mural cells of the central nervous system (CNS). SARS-CoV-2 may directly destroy pericyte and further reduce blood supply to the brain, and ultimately result in neuronal dysfunction 90, 91. Pericytes are perivascular cells within the brain that are proposed as SARS-CoV-2 infection points 92. Moreover, the infection of olfactory bulb pericytes may disrupt neuronal signals through local inflammation and cytokine release, which are related to the functional impairment caused by olfactory bulb vascular injury and hypoperfusion 92. A recent study shows that SARS-CoV-2 could preferentially infect astrocyte over other cells 93. They exposed the brain organoids to SARS-CoV-2 and found that almost only astrocytes were infected. Similarly, Daniel Martins-de-Souza *et al.* analyzed the brains of 26 COVID-19 deceased, among 5 samples in which the brain cells were infected with the SARS-CoV-2, up to 66% of the infected cells were indeed astrocytes 94.

Severe neurological complications in COVID-19 are both rare and variable in nature. Indeed, any part of neuraxis seems to be susceptible to damage by SARS-CoV-2. Neurological disorders may result from systemic cardiopulmonary failure and metabolic abnormalities caused by infections, direct viral invasion, or viral autoimmune reactions. In an unpublished report from Wuhan, China, 78 (36.4%) of 214 hospitalized COVID-19 patients had some form of neurological disorder, the most common features of which were dizziness, headache, hypogeusia, and hyposmia 95. While serious neurological complications have been reported in patients with otherwise mild COVID-19 96, the most severe complications occur in critically ill patients and are associated with significantly higher mortality 83, 97. And neurological disorders were found to be more common in critically ill patients, such as stroke in 6 (2.8%), impaired consciousness in 16 (7.5%), and muscle injury in 23 (10.7%). These were subdivided into those thought to reflect CNS and peripheral nervous system (PNS) 95.

#### 2.4.1. Central nervous system dysfunction

##### 2.4.1.1. Dizziness and headache

Headache is a possible symptom in any systemic viral infection such as SARS-CoV-2. In COVID-19, headache usually coincides with fever. Headache was reported in 6.5-14.1% of COVID-19 cases 73, 98, 99. However, the prevalence of headaches in COVID-19 infection seems to be underestimated in terms of variety and clinical description. According to this finding raised by Dr. Robert Belvis 100, headaches related to COVID-19 can be classified in the 2 phases of the disease. Acute headache, primary cough headache, tension type headache, and heterologous headache caused by systemic viral infection can appear in Phase I (influenza like phase) and hypoxia and headache caused by new onset headache can occur in Phase II (cytokine storm phase with an increase of IL-2, IL-6, IL-7, IL-10, TNFα, G-CSF, IFN-γ inducible protein 10, MCP1, and macrophage inflammatory protein 1-α). Headache may be associated with cytokine storm in SARS-CoV-2 infection, but further research is needed to better understand this link.

##### 2.4.1.2. Acute cerebrovascular disease

With the spread of COVID-19 around the world, there is more and more evidence related to cerebrovascular diseases and other forms of vascular diseases. COVID-19 cerebrovascular disease appears to be predominantly ischemic and involves large vessels. In the elderly, it reflects the underlying severity of the systemic disease as well as the hyperinflammatory state, whereas in the younger patients, it seems to be due to hypercoagulopathy 101, 102. Besides hypercoagulable state, SARS-CoV-2 can also infect and damage endothelial cells. However, it remains to be determined whether the endothelial cell damage caused by the virus or even the real vasculitis will lead to the cerebrovascular syndrome associated with COVID-19, which will require more detailed angiography and neuropathological analysis.

Stroke may have significant interaction with COVID-19, and stroke is not uncommon among patients hospitalized with COVID-19. Several studies have reported strokes in COVID-19 patients, with rates ranging from 1%-3% and up to 6% of critically ill patients 5, 73, 99, 103. These patients may develop more severe coagulopathy, defined as COVID-19-related coagulopathy, which may be caused by inflammation, including inflammatory cytokine storms. It is not clear whether these strokes are caused by SARS-CoV-2 or the incidence rate of stroke in these high-risk groups, and these high-risk groups also happened to have SARS-CoV-2. SARS-CoV-2 infection does play a role in stroke, which is reasonable, because infection usually increases the risk of stroke. Therefore, understanding the relationship between infection and stroke has taken on urgency in the era of the COVID-19 pandemic. The association of infection and stroke is also bidirectional. Furthermore, ACE is known for its role in blood pressure regulation through the renin angiotensin aldosterone system (RAAS), and it can also play a role in fertility, immunity, hematopoiesis and obesity, fibrosis and Alzheimer's dementia 104. More importantly, it is a functional receptor of SARS-CoV-2 105. Therefore, understanding the interaction between SARS-CoV-2 and ACE2 is very important for the design of therapy for this disease. In the case of severe infection, myocardial injury and arrhythmias, such as atrial fibrillation, can lead to cardiac embolism and cerebral infarction 106. In addition to primary viral diseases, a considerable number of critical patients with COVID-19 may also have secondary bacteremia. In one case series, about 10% of patients requiring mechanical ventilation have bacteremia, which increases the risk of stroke by more than 20 fold 107. Septic cerebral emboli often lead to hemorrhage. In a postmortem magnetic resonance imaging (MRI) study, 10% of brain showed signs of hemorrhage 108. Emerging evidence suggested the role of infection, as a contributor to long-term risk of atherosclerotic disease and stroke; immune dysregulation after stroke and its effect on the risk of stroke-associated infection; and the impact of infection at the time of stroke on the immune reaction to brain injury and subsequent cognitive decline 109. In conclusion, these clinical findings suggest that SARS-CoV-2 may have adverse effects on brain through a variety of pathophysiological pathways and eventually lead to vascular brain injury.

#### 2.4.2. Peripheral nervous system

##### 2.4.2.1. Peripheral organ dysfunctions

COVID-19 also damages other organs, and metabolic and pathological evidences on COVID-19-induced renal, cardiac, hepatic, gastrointestinal and endocrine organ damage have been presented so far 106, 107, 110, 111. The resulting systemic metabolic changes, including water and electrolyte imbalance, hormonal dysfunction, and accumulation of toxic metabolites, may also contribute to some of the nonspecific neurological manifestations of the disease, like confusion, agitation, headache, cardiac involvement, which may affect brain by reducing cerebral perfusion. The lung is the most seriously affected organ in COVID-19, accompanied by a large number of alveolar injuries, edema, inflammatory cell infiltration, microvascular thrombosis, microvascular injury and bleeding 112. SARS-CoV-2 was mainly detected in lung cells and epithelial progenitor cells 112, 113. Severe hypoxia (acute respiratory distress syndrome, ARDS) caused by respiratory failure caused by lung injury requires auxiliary ventilation 114. Consistent with hypoxic brain damage, the autopsy study of COVID-19 showed that neuronal damage was found in the most vulnerable areas of brain, including neocortex, hippocampus and cerebellum 84, 115.

##### 2.4.2.2. Neurological autoimmune disorders with COVID-19 on PNS

Viral illnesses may trigger an autoimmune response, which affects the central or peripheral nervous system. As mentioned earlier, acute inflammatory demyelinating peripheral neuropathy (AIDP)/Guillain-Barre syndrome (GBS) may be a consequence of infection of peripheral nervous system. MERS also causes post infectious brainstem encephalitis and GBS 116. Reports of SARS-CoV-2 transverse myelitis also began to appear 73. GBS is an acute polyradiculopathy characterized by rapid progressive symmetrical limb weakness, sensory disturbance during examination, and facial weakness in some patients. 11 patients had GBS with weakness of all four limbs with or without sensory loss 117-119, of which three patients only had a paralytic variant with leg weakness 118, 120, 121, and one had lower limb paresthesia 118.

### 2.5. COVID-19 on motor system

Motor complications with COVID-19 such as critical illness myopathy, polyneuropathy, GBS, Bell's palsy, and Parkinson's disease (PD) have been reported recently 122.

#### 2.5.1. Autonomic dysfunction preceding acute motor axonal neuropathy (AMAN)

A 20-year-old patient had previous autonomic dysfunction characterized by sinus arrhythmia, postural hypotension, intermittent sweating, constipation, erectile dysfunction, and chest crush 123. This is unique in this case, except for the fact that autonomic dysfunction precedes motor weakness, which is very rare. Since the disease associated with COVID-19 was mild and almost asymptomatic, the patient was only treated with acetaminophen. For AMAN, intravenous immunoglobulin (0.4 g/kg/day, for 5 consecutive days) was started. After 15 days of treatment, the patient's dyskinesia and autonomic nervous system began to improve. After rigorous physical therapy, the patient was able to walk with some help one month after admission.

#### 2.5.2. Oculomotor nerve palsy and motor peripheral neuropathy

A report described a 65 year old man with oculomotor nerve palsy 124. He had a 5-day history of persistent diplopia and left blepharoptosis. MRI and Magnetic resonance angiography (MRA) showed no abnormalities, but computed tomography (CT) of chest showed diffuse ground glass opacity. SARS-CoV-2 was detected in throat swabs. Importantly, a case reported that a 69 year old man was admitted to the COVID-19 ward with bilateral weakness of lower limbs three days before admission 120. Due to chronic cough, he underwent a COVID-19 swab test in the emergency room and was admitted to the COVID-19 ward. The strength of his bilateral knee extension was reduced by four fifths, the strength of other muscle groups was normal, his knees and ankles had no convulsions, and his gait was ataxia. This case is different from the case of GBS reported in the Journal of Neurology of the Lancet. There is microbiological evidence of COVID-19 infection at the time of admission, and influenza like symptoms appear only 7 days after the symptoms appear. He had distal weakness and hyporeflexia, no back pain or sensory level, suggesting motor peripheral neuropathy. However, this diagnosis still needs further characterization and analysis. At present, the physical condition of the case does not allow us to carry out further characterization.

#### 2.5.3. Bell's palsy

Wan *et al*. described the first case of Bell's palsy in a 65 year old woman who developed left lower motor neuron facial paralysis 2 days after mastoid pain. Interestingly, the patient had no symptoms of other viral diseases 125. MRI showed no abnormality, but CT showed a piece of ground glass shadow in the right lower lung, which was suspected to be SARS-CoV-2. SARS-CoV-2 infection was confirmed by real-time reverse transcription polymerase chain reaction (RT-PCR).

#### 2.5.4. Parkinson's disease and motor symptom

Patients with underlying neurological dysfunction, such as PD, often have associated cardiovascular and respiratory disorders, which increase their risk of developing severe COVID-19. In addition, due to fever and altered dopaminergic drug intake, PD patients with COVID-19 have developed Parkinson's disease high fever syndrome, a motor disorder emergency 126. Although patients who experience this phenomenon may recover from COVID-19, some patients may leave significant disabilities, while others may not survive 127. Recently, people with or without PD participating in the online study Fox Insight (FI) were invited to complete a survey to assess COVID-19 symptoms and the pandemic's effect. The report showed that people with PD and COVID-19 experienced new or worsening motor (63%) symptom. And people with PD but not diagnosed with COVID-19 reported disrupted medical care (64%), exercise (21%), and social activities (57%), and worsened motor (43%) symptoms 128. These results suggested that during COVID-19 infection, people with PD reported worsening of many PD-related motor and non-motor symptoms, including rigidity, tremor, walking difficulties, emotional symptoms, cognition and fatigue.

### 2.6. COVID-19 on urinary system

While most COVID-19 patients present with mild symptoms, a small percentage can gradually develop acute respiratory distress syndrome and multiple organ dysfunction syndromes, leading to death 129. Because these symptoms may overlap with other common disease processes, it is difficult to identify these symptoms as the underlying cause directly related to COVID-19. Recently, Mumm and his colleagues reported increased frequency of urination, and identified this in seven males out of 57 patients currently being treated in COVID-19 wards 130. In the absence of any other cause, urinary frequency may be secondary to viral cystitis due to the underlying COVID-19 disorder. We recommend considering urinary frequency as a memory tool for patients with infectious symptoms to increase urologists' awareness during the current COVID-19 pandemic, preventing the fatal effects of erroneous interpretation of urinary symptoms.

#### 2.6.1. Lower urinary tract symptoms

One of the most frequently reported epidemiological data is gender related COVID-19 mortality. Studies conducted in various countries have shown that men are more susceptible to COVID-19 infection. Male patients accounted for 73% of deaths in China, 59% of deaths in Korea, and 70% of patients who died in Italy were male 131, 132. In addition, a recent review of current epidemiological studies of 59254 patients from 11 different countries suggested an association between male sex and higher mortality 133. In addition, experimental studies performed by Channappanavar and his colleagues showed that male rats were more susceptible to SARS-CoV infection than age-matched female rats 134. Epidemiological studies of COVID-19 are essential to better understand the pathogenic mechanisms of the disease and to identify good treatment strategies. Lower urinary tract symptoms (LUTS) are a term that encompasses a wide range of symptoms that occur after storage and voiding. LUTS is common in adult men and is often associated with benign prostatic hyperplasia (BPH) 135. The prevalence of BPH increases significantly with age, and BPH-related LUTS often emerged as a natural result of aging and androgen exposure. Prostatic hyperplasia increases urethral resistance, leading to compensatory changes in bladder function. Because bladder outlet resistance increases, detrusor pressure also increases with increasing urine flow. Increased detrusor resistance also causes LUTS by affecting bladder storage function 136. LUTS was assessed by approved questionnaires, such as the International Prostate Symptom Score (I-PSS), and the results indicated that LUTS could effectively predict the severity of COVID-19.

#### 2.6.2. Acute kidney injury

Acute kidney injury (AKI) has been reported in up to 25% of critically ill patients with SARS-CoV-2 infection, in particular in those with serious infections, and has been associated with substantial morbidity and mortality 137, 138. In most studies, AKI develops throughout hospitalization, with a mean of 5 to 9 days after admission 139, 140. AKI develops more frequently in patients with the most severe diseases (especially ARDS, requiring invasive mechanical ventilation), including elderly patients or those with hypertension or diabetes. The causes of kidney involvement in COVID-19 may be multifactorial, and cardiovascular comorbidity and predisposing factors (such as sepsis and nephrotoxin) are important factors. However, renal tubular injury is common and associated with the cytopathic effect of renal resident cells and cytokine storm syndrome 141, 142.

### 2.7. COVID-19 on reproductive system

**Figure 4:**
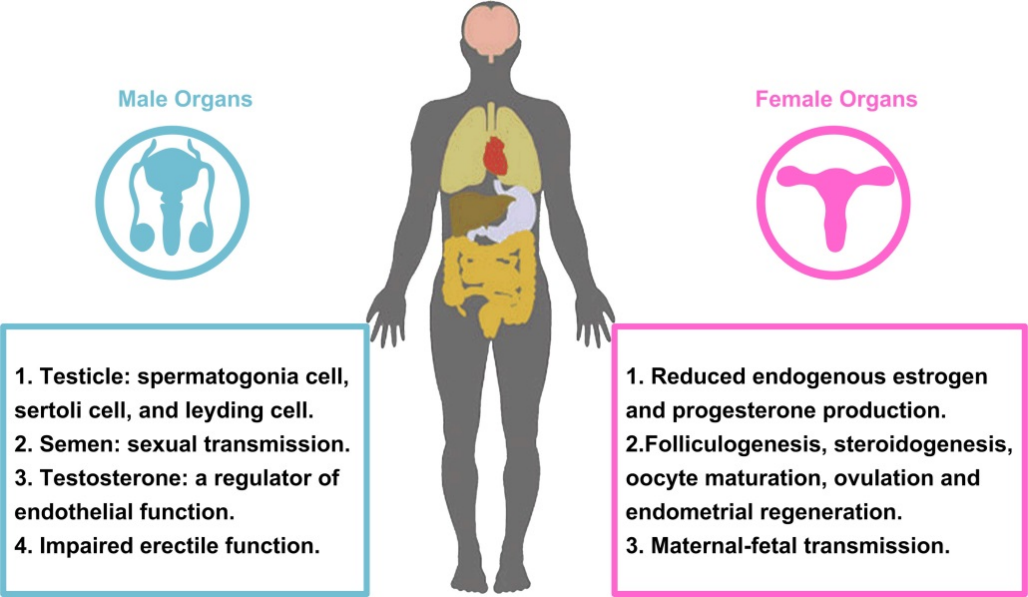
Effects of COVID-19 on the male and female reproductive systems.

Since the emergence of the SARS-CoV-2 infection in December 2019, it has rapidly spread across all over the world. Additionally, it has been demonstrated that SARS-CoV-2 infection not only damage to respiratory system, but other organs of human, such as heart, liver, oesophagus, kidney, bladder, and ileum 143. As mentioned above, ACE2, the functional receptor for SARS-CoV-2, modulates the cleavage of Ang II and Ang 1-7. Because SARS-CoV-2 enters cells by binding to ACE2 receptor, the reproductive cells and/or tissues expressing ACE2 may be susceptible to virus infection, and their functions may be interfered theoretically. ACE2, Ang II and Ang 1-7 can regulate the basic functions of male and female reproductive system. In the female, it includes folliculogenesis, steroidogenesis, oocyte maturation, ovulation and endometrial regeneration 144, 145. In the male, testicular ACE2 may regulate testicular function, play a role in sperm function, and may affect sperm contribution to embryo quality 146, 147. An important and interesting topic in the era of COVID-19 is the ability of virus to affect male and female reproductive capacity (Fig. 4), and whether pregnant women with COVID-19 have an increased risk of death or comorbidity.

#### 2.7.1. Reproductive hormones

One of the main functions of ovary and testis is steroid production. Therefore, the evaluation of sex hormone levels can provide the evaluation of gonadal function in patients with COVID-19. Ma *et al*. compared sex related hormone levels in 119 reproductive men infected with SARS-CoV-2 with 273 age-matched controls 148. Most patients have moderate to severe diseases. The serum luteinizing hormone (LH) was increased and serum Testosterone (T)/LH ratio was decreased in the COVID-19 group. Rastrelli *et al*. found that the deterioration of clinical condition is accompanied by the gradual decrease of T level and the increase of LH level 149. However, these results should be interpreted with caution, as pre-infection sex hormone baselines are not available for these patients. In addition, hypogonadism is a common systemic disease. In the case of COVID-19, it is not clear whether the low T level observed is the result of the direct effect of COVID-19 on gonadal function 150. In the female, severe acute illness may alter hypothalamic pituitary gonadal (HPG) axis function, reducing endogenous estrogen and progesterone production 151.

#### 2.7.2. Sex and COVID-19

ACE2 receptor is more abundant in male reproductive system than in female reproductive system. ACE2 was low expressed in oviduct (ciliated cells and endothelial cells), ovary, vagina, cervix and endometrium 152, 153. On the other hand, the expression of ACE2 was the highest in testis, high in leydig cells and sertoli cells, and medium in seminal vesicle cells 154, 155. Therefore, it is expected that testis is more vulnerable to SARS-CoV-2 infection than ovary.

##### 2.7.2.1. Male

As we all know, ACE2 not only expresses in the lung, but also extensively expresses in spermatogonia, sertoli and leydig cells in testicle. Indeed, it has been found that the testis may be infected with SARS-CoV-2, which may lead to further reproductive system diseases 156, 157. Similarly, it is well known that viruses can enter testicular cells, cause viral orchitis, and even lead to male infertility and testicular tumors 158, 159. In ACE2 positive cells, the abundance of transcripts related to SARS-CoV-2 replication and transmission was higher than that related to male gametogenesis. The expression of ACE2 in human testis suggests that SARS-CoV-2 may infect male gonads and lead to male reproductive dysfunction. We know that temperature is very important for cell growth and development, and 37°C is the best temperature for cell growth. However, almost all SARS-CoV-2 infected people have persistent fever. When the human body is in a state of high fever for a long time, the testicular temperature will change, and the germ cells will be damaged and degenerated 160, 161. Furthermore, several results showed that SARS-CoV-2 existed in semen and testis of SARS-CoV-2 infected patients in acute and convalescent stages. Therefore, it also supports the result that SARS-CoV-2 can be sexually transmitted through men 162, 163. SARS-CoV-2 infection can cause systemic local inflammation, and due to the imperfect blood testis/vas deferens/epididymis barrier, SARS-CoV-2 may propagate to the male reproductive tract 164. However, the virus cannot replicate in the male reproductive system, it may persist, and this phenomenon may be due to the special immunity of testis 165, 166. These results suggest that sexual transmission may be an important link in the prevention of transmission. A recent report on 31 Italian male COVID-19 patients noted that some patients developed hypergonadotropic hypogonadism after disease onset 149. In this study, low levels of serum testosterone (total and free) may serve as a predictor of poor outcome in SARS-CoV-2-infected men. Testosterone, as a regulator of endothelial function, inhibits inflammatory responses by increasing the levels of anti-inflammatory cytokines, such as IL-10, and decreasing the levels of pro-inflammatory cytokines, such as TNFα, IL-6, and IL-1β 167, 168. Therefore, it can be hypothesized that suppressed testosterone levels may be one of the reasons for the large differences in mortality and hospitalization between men and women and may also explain why SARS-CoV-2 most commonly infects elderly men. As mentioned earlier, patients with hypogonadism have higher concentrations of TNFα, IL-6 and IL-1β due to reduced inhibition. This eventually worsens endothelial dysfunction and further impairs erectile function. While erection is certainly a trivial matter for patients in ICU, there is reason to suspect that impaired vascular function may persist among survivors of COVID-19 and even become a public health problem in the coming months. In addition, since erectile function is a predictor of heart disease 169, 170, investigating whether erectile dysfunction occurs in patients with COVID-19 may also be a good alternative indicator of general cardiovascular function, improving patient care and quality of life. In addition, only a small retrospective study assessed the presence of SARS-CoV-2 in prostate secretions. One study evaluated prostate secretions from 18 male patients with confirmed COVID-19 and 5 suspected cases, and found that no SARS-CoV-2 RNA expression was detected in the samples of all patients assessed 171. As any kind of sudden disease, there are more doubts and hypotheses, rather than certainty, about the impact of COVID-19 on male reproductive system. Many studies have been carried out to better understand the disease and its short- and long-term effects on health. As has been demonstrated in other viral diseases, the involvement of male reproductive system is a possibility, which may reveal a new route of transmission and/or impact on its function. The virus has been found in the semen of infected patients, but its impact on male reproductive health remains to be further investigated.

##### 2.7.2.2. Female

Studies have shown that ACE2 mRNA is highly expressed in the ovaries of women of childbearing age and postmenopausal women. These results implied that female reproductive system may be at risk of SARS-CoV-2 infection 172. Single cell sequencing was used to analyze the expression of ACE2, TMPRSS2, cathepsin B and L (CTSB and CTSL, respectively) in human ovarian cells 173. It was found that the expressions of ACE2 in stromal cells and perivascular cells of ovarian cortex were very low. TMPRSS2 was not expressed in different types of oocyte nest cells, while CTSB and CTSL were expressed in all ovarian cells. There was no co-expression of ACE2/CTSB and ACE2/CTSL in all ovarian cell types. Because ACE2 needs the co-expression of protease TMPRSS2 or CTSB/L to make protein on its surface and to ensure enter host cells. The expression rate of ACE2 in ciliated cells, secretory cells and leukocytes was less than 5%. On the contrary, the expression levels of protease TMPRSS2 and CTSL/B were different in different fallopian tube cells, but CTSL was not detected in any fallopian tube cells. No co-expression of ACE2, TMPRSS2 or CTSB was observed in any oviduct cells.

Furthermore, studies have shown that 70 pregnant women infected with SARS-CoV-2 had symptoms such as fever (84%), cough (28%), and dyspnea (18%). Obstetric complications included preterm delivery (39%), intrauterine growth restriction miscarriage (10%) and miscarriage (2%). Amniotic fluid, cord blood, throat swabs and neonatal milk were collected from 9 pregnant women with SARS-CoV-2 infection in Wuhan, China, and the results indicated that there was no direct evidence of vertical transmission of SARS-CoV-2 174, 175. But it was found in additional studies that ACE2 has transient overexpression and increased activity during pregnancy, particularly in the placenta, implying that there may be vertical transmission. Previous clinical studies have not observed evidence of vertical transmission of SARS-CoV-2 among cases, a phenomenon that still needs to be more carefully investigated in clinical practice 176.

SARS-CoV-2 may infect ovary, uterus, vagina and placenta through the universal expression of ACE2. In addition, SARS-CoV-2/ACE2 may interfere with female reproductive function, leading to infertility, menstrual disorders and fetal distress 177. We recommend following up and evaluating fertility after recovery from SARS-CoV-2 infection and, if possible, postponing pregnancy, especially in young women. Moreover, we should continue to pay attention to the situation of pregnant patients and fetus, and take timely measures. In order to reduce the incidence of SARS-CoV-2 infection, special care was given to healthy pregnant women, puerpera and newborns.

COVID-19 sex differences in incidence, comorbidities, and mortality males are at higher risk and require prompt action to understand the sources of biological and behavioral differences. As the impact of SARS-CoV-2 on male/female reproductive system becomes more intensively studied, we will control and prevent the SARS-CoV-2 infection system in the male/female reproductive system more and more effectively.

### 2.8. COVID-19 on digestive system

At the initial stage of COVID-19 pandemic, a series of respiratory manifestations caused by the virus are the first to be discovered and concerned. Thence, SARS-CoV-2 is initially considered to be most likely to cause respiratory illnesses and spread from human to human mainly via respiratory tract. Since SARS-CoV-2 RNA in stool specimen was first reported in the study of the first case of COVID-19 infection in USA 178. Ongoing reports of viral gastrointestinal infection and fecal-oral transmission of the virus are a matter of widespread concern.

#### 2.8.1. The impact of SARS-CoV-2 on gastrointestinal tract

**Figure 5:**
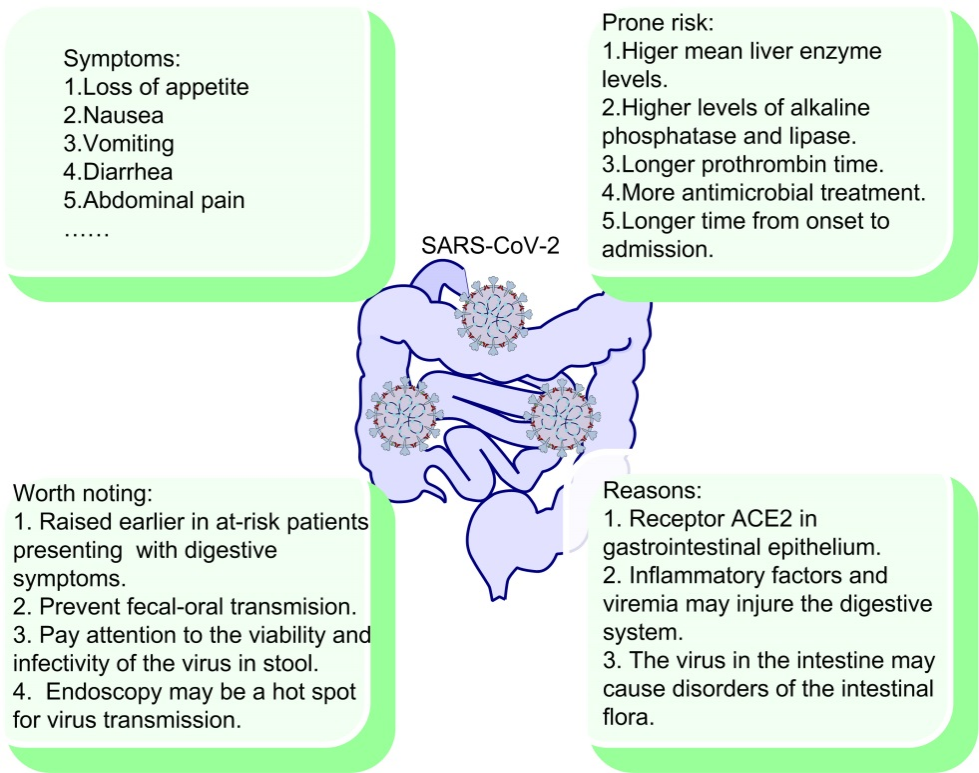
SARS-CoV-2 infection of the gastrointestinal tract.

The digestive system has been reported not only as a site of disease expression, but also as a possible driver of disease severity and viral transmission. Several studies have reported a high prevalence of gastrointestinal symptoms in patients infected with SARS-CoV-2, mainly including loss of appetite, nausea, vomiting, diarrhea and abdominal pain 178-180 (Fig. 5). Importantly, multiple studies have confirmed a fraction of COVID-19 patients only experience abdominal symptoms without fever or respiratory manifestations 179, 180. Therefore, it is important that clinicians should maintain a high index of vigilance in patients with gastrointestinal symptoms. In addition, SARS-CoV-2 appears to persist in patients' stool specimen, even though the patients' throat swabs become negative 181, 182. The study also concluded that patients with gastrointestinal symptoms have significantly longer time from onset to hospital admission than patients without gastrointestinal symptoms 180. In contrast, a study reported a low incidence (3.8%) of gastrointestinal symptoms 67. Of course, due to the difference in medical record samples and symptom ascertainment, different conclusions are normal. However, the risk of virus transmission here is unanimously recognized and cannot be ignored.

Endoscopic and histologic findings give us a more comprehensive understanding of the impact of SARS-CoV-2 on the gastrointestinal tract. In the study, a patient with COVID-19 present upper gastrointestinal bleed 181. Gastrointestinal endoscopy was performed and mucosa damage in the esophagus was observed 181. Additionally, the hematoxylin-eosin staining results in this study showed that the mucous epithelium of esophagus, stomach, duodenum, and rectum did not appear obvious damage 181.

What's more, the incidence of liver injury in COVID-19 patients is 39.6% to 43.4%, which is mainly manifested by increased levels of alanine aminotransferase (ALT) and aspartate aminotransferase (AST), as well as hypoalbuminemia 183, 184. In contrast, some other studies did not find significant liver injury in COVID-19 patients 180, 185. However, the study also reported that patients with digestive symptoms had higher mean liver enzyme levels and longer prothrombin time than those without digestive symptoms, which could reflect the potential risk of liver injury 185. It is hard to explain the variations in liver test abnormalities among those studies, thus the effect of SARS-CoV-2 on liver needs further research. More importantly, another study showed that patients with gastrointestinal symptoms were reported more likely to suffer liver injury due to their elevated ALT and AST compared with patients without gastrointestinal symptoms 180.

#### 2.8.2. Why gastrointestinal tract occurs?

There are many proposed reasons for the occurrence of viral gastrointestinal infection. First, gastrointestinal epithelial cells has been shown to express ACE2, the receptor of SARS-CoV-2 181. Moreover, the positive staining of ACE2 and SARS-CoV-2 was also observed in gastrointestinal epithelium from other patients who tested positive for SARS-CoV-2 RNA in feces 181. Second, SARS-CoV-2 damages the digestive system through an inflammatory response. The intestine is the largest immune organ in the human body, and COVID-19 patients present high inflammatory level 186. The chain reaction of inflammatory factors and viremia may injure the digestive system. Finally, the intestinal flora plays an important role in body. The number of intestinal flora in the human intestine is astonishing and diverse, which is very important for the normal functioning of digestive system. The virus in the intestine may cause disorders of intestinal flora, which result in digestive symptoms 187. Several studies have also reported dysbiosis of intestinal flora in COVID-19 patients 188, 189. In a study, the author observed that gut virome and bacteriome in the COVID-19 patients are notably different from those of the healthy control, and this difference also exists between patients of different severity 188. The study also confirmed the virome differences and bacteriome dysbiosis in mouse COVID-19 model. More importantly, the study reported the differential expression of immune/infection-related genes in mouse intestinal epithelial tissues during infection, such as the polymeric immunoglobulin receptor (PIGR), interleukin-15 (IL-15), and tribbles pseudokinase 1 (TRIB1) 188. Therefore, SARS-CoV-2 may cause the changes in the expression of certain genes in gastrointestinal tissues, which may be related to the occurrence of gastrointestinal symptoms. In conclusion, the correlation between gastrointestinal symptoms and patients' symptoms, diagnosis, treatment, and outcomes have not been fully elucidated. It is important and worthy for us to keep exploring.

## 3. Treatment of COVID-19

The outbreak of the COVID-19 pandemic has plunged the world into an unprecedented crisis. The virus quickly swept across the globe, causing enormous loss of life, destroying the livelihoods of billions of people and endangering the global economy 190. The cumulative number of confirmed cases of COVID-19 worldwide has not yet peaked and the situation remains serious, so the United Nations is leading and coordinating global efforts to support countries in their efforts to combat the pandemic. However, up to now, there is still no good effective treatment for COVID-19 191. Therefore, it has forced many countries and regions around the world to quickly carry out the research of the novel coronavirus.

Particularly, the development of preventive vaccine against SARS-CoV-2 is the key to control and prevent the outbreak of a pandemic 192. According to the research progress of the COVID-19 vaccine updated by the World Health Organization, so far there are 81 new coronavirus vaccines in the clinical development stage, and more than 180 vaccines are in the preclinical development stage 193. Simultaneous research and development of multiple vaccines will ensure the quality of the vaccine. The acceleration of scientific research and clinical trials and the granting of emergency use authorization by the relevant government will enable the vaccine to be put on the market as soon as possible to deal with the sudden spread of the COVID-19. In addition to vaccines, an important strategy for the control and treatment of COVID-19 is to modulate the immune system using other methods, including the plasma therapy, suppressing inflammatory cytokines, kinases inhibitors, cell-based therapies, complement therapy, monoclonal antibody therapy and immune potentiator (Table 1), which are the key immunotherapeutic approaches to deal with COVID-19.

### 3.1. Plasma therapy

It has been demonstrated that convalescent plasma from COVID-19 patients that have recovered from the SARS-CoV-2 infection can be utilized as therapy for patients with COVID-19, without severe adverse events 194. Plasma therapy works by passive transfer antibodies to neutralize the virus. Clinical data suggested that patients treated with convalescent plasma had lower mortality than those who were not 195. However, this method requires the collection of plasma from a sufficient number of convalescent patients. Because of this limitation, plasma therapy is considered as an option for the treatment of patients with severe COVID-19. Importantly, during the progression of the COVID-19 disease, the quality of neutralizing antibodies in convalescent plasma samples has changed, and different plasma samples exhibited different antiviral potentials. Therefore, it is necessary to estimate the function and titer of neutralizing antibodies from the donors before treatment 196.

### 3.2. Cytokine inhibition

The patients with severe COVID-19 were more likely to generate the cytokine storm, leading to tissue damage and multi-organ failure. A huge release of IL-1β, IL-2, IL-6, IL-10, GM-CSF, TNFα, and MCP-1 resulted in immune dysregulation. Therefore, the use of immunomodulators is beneficial to regulate the imbalanced immune responses. Current studies revealed the role of IL-6 in the pathogenesis of COVID-19, and the development of drugs targeting the IL-6 pathway is promising for relieving inflammation in COVID-19 patients 40. Tocilizumab, a monoclonal antibody targeting the IL-6 pathway, has been approved to treat COVID-19. After treatment with Tocilizumab, the clinical manifestations of patients have been improved, including rapid control of fever and improved respiratory function 197. Similarly, monoclonal antibodies available for COVID-19 therapy are Anakinra, Etanercept, Mavrilimumab, and Bevacizumab, which target the IL-1R, TNFα, GM-CSF, VEGF, respectively, and have been summarized in Table 1.

### 3.3. Kinase inhibitors

Janus associated protein kinase (JAK) is a potential therapeutic target for controlling SARS-CoV-2 infection. Baricitinib, upadacitinib, fedratinib, and ruxolitinib, as JAK inhibitors, have been approved for treating rheumatoid arthritis and multiple inflammatory diseases. Currently, a randomized, double-blind, placebo-controlled, parallel-group Phase III clinical trial of baricitinib in COVID-19 patients is ongoing, the aim of which is to investigate whether the baricitinib is effective in COVID-19 hospitalized patients (NCT04421027). Bruton tyrosine kinase (BTK) inhibitors are another group of tyrosine kinase inhibitors. AstraZeneca initiated a randomized, global clinical trial to evaluate the efficacy of the acalabrutinib, one of BTK inhibitors, in the treatment of patients with COVID-19 accompanied by cytokine storm (NCT04497948). Other kinase inhibitors available for COVID-19 treatment, including sunitinib, a receptor tyrosine kinase (RTK) inhibitor, and erlotinib, an epidermal growth factor receptor (EGFR) tyrosine kinase inhibitor, were shown to block SARS-CoV-2 entry 198.

### 3.4. Cell-based therapies

Accumulating evidence has revealed that the number of NK cells in peripheral blood was significantly decreased and most of them displayed a functional exhaustion phenotype in COVID-19 patients. With this in mind, the lack and exhaustion of NK cells may be one of the reasons for the unrestricted progression of COVID-19. CYNK-001 is the allogenic, human placental hematopoietic stem cell-derived NK cells that can recognize and kill the virus-infected host cells. A Phase I/II clinical trial is evaluating its safety, tolerability, and efficacy in COVID-19 patients (NCT04365101). Another Phase I/II clinical trial in patients with COVID-19 is ongoing, which aims to investigate the efficacy of the constructed NKG2D-ACE2 CAR-NK cells derived from cord blood in treating severe and critical COVID-19 (NCT04324996).

Mesenchymal stem cells (MSCs) are a population of multipotent stem cells with high potential ability of self-renewal, proliferation, multi-directional differentiation and immunomodulatory 199. MSCs have an immunosuppressive effect on different immune cells, such as T cells, B cells, NK cells and DCs, through producing a large number of immunosuppressive agents, including indoleamine-pyrrole 2, 3-dioxygenase (IDO), prostaglandin E2 (PGE2) and IL-10. Several studies have reported that MSCs served as a treatment against COVID-19-related cytokine storm and lung injury. The clinical trials of seven patients with severe COVID-19 has confirmed the efficacy and safety of intravenous administration of MSCs resulting from increasing lymphocyte and anti-inflammatory cytokines (IL-10) and decreasing pro-inflammatory cytokines, such as C-reactive protein (CRP) and TNF 200.

The balance between effective T cells (Teffs) and Tregs in the adaptive immune response is most likely a major factor influencing the outcome of COVID-19 51, 201. A clinical study aimed to analyze the global T cell receptor (TCR) repertoire of peripheral blood derived Tregs and Teffs, to better understand the nature of Tregs and Teffs against COVID-19, and to reveal biomarkers associated with disease severity (NCT04379466). This is great meaningful for understanding the pathophysiology of the disease and designing therapeutics and vaccines.

### 3.5. Monoclonal antibody therapy

Monoclonal antibodies, which accurately identify and destroy antigens, play an important role in disease diagnosis, anti-infection, and anti-tumor. For viral infections, a neutralizing monoclonal antibody can specifically neutralize the virus and prevent the virus from entering the cell to proliferate. Thus, neutralizing monoclonal antibodies are regarded as one of the most promising options for the prevention and treatment of COVID-19. At present, the SARS-CoV-2 S protein-targeting monoclonal antibodies are mainly the receptor binding domain (RBD)-targeting antibodies 202-205. Bamlanivimab (LY-CoV555) is a neutralizing monoclonal antibody that binds to the RBD of SARS-CoV-2 S protein, and has been shown in Phase II trial to significantly reduce SARS-CoV-2 levels in patients 202. According to the U.S. Food and Drug Administration (FDA) announcement, Bamlanivimab was the first monoclonal antibody which receive the emergency use authorization (EUA) on November 9, 2020. Etesevimab (LY-CoV016) is another neutralizing monoclonal antibody that also binds to the RBD of SARS-CoV-2 S protein 203.The combination therapy of Bamlanivimab and Etesevimab accelerated the decline of SARS-CoV-2 viral load and reduced the mortality rate of COVID-19 patients 203, 206, and obtained the EUA granted by FDA on February 9, 2021. Unfortunately, whether it was the use of Bamlanivimab alone or the combined treatment of Bamlanivimab and Etesevimab, these have been shown to be unable to resist the mutant virus. REGEN-COV, combination monoclonal antibodies of Casirivimab (REGN10933) and Imdevimab (REGN10987) which bind to the RBD of SARS-CoV-2 S protein 204, 205, has also obtained the EUA from FDA on November 21, 2020, and more importantly, REGEN-COV still retains its effectiveness against a variety of mutant viruses 205. Sotrovimab (VIR-7831), a monoclonal antibody which also binds to the RBD of SARS-CoV-2 S protein has shown that the risk of hospitalization or death in the Sotrovimab group was 85% lower than that of the control group 207, and Sotrovimab obtained the EUA from FDA on May 26, 2021. Additionally, 4A8, as an N-terminal domain (NTD)-targeting antibody, has a strong virus neutralization ability 208. Combination of the NTD-targeting antibody with RBD-targeting antibody may avoid the escaping mutations of the virus and serve as promising “cocktail” therapeutics. Of course, there are many other monoclonal antibodies for the treatment of COVID-19 in the research and development stage or clinical trial stage, and it is of great significance and contribution to the fight against the global epidemic.

### 3.6. Other therapies

An adjunctive therapy available for COVID-19 is cytosorb, which absorbs a broad spectrum of cytokines, damage-associated molecular patterns (DAMPs), and pathogen-associated molecular patterns (PAMPs) in the blood circulation to reduce inflammation and improve immunopathology of the disease 209. Complement inhibitors have emerged as the drug candidates against SARS-CoV-2 infection. In a cohort study, COVID-19 patients were treated with the eulizumab and the cyclic peptide AMY-101 to block complement C5 and C3, respectively, and it was shown that complement inhibition alleviated hyper-inflammation characterized by a significant decrease in serum IL-6 and CRP, reduced neutrophil counts, and markedly improved lung function and lymphocyte recovery 210. Because the surface ligands of SARS-CoV-2 are constantly altered to escape neutralizing antibodies, one strategy is to apply drugs that block receptors for such ligands on host cells, such as ACE2. Immune potentiator treatment strategies aim to stimulate innate and adaptive immunity through multiple mechanisms to eliminate viral infections. These potentiators include antimicrobial peptides, immune checkpoint inhibitors, pattern recognition receptor (PRR) ligands, and signaling compartments 211. Neutralizing antibodies against PD-1, alone or in combination with thymosin, are under investigation for their efficacy in COVID-19 cases (NCT04268537). Additionally, the use of corticosteroid to treat patients with COVID-19 remains controversial currently, and relevant clinical trials are ongoing (NCT04244591).

## 4. Conclusions and Perspectives

Similar to SARS, COVID-19 manifests mainly as the symptoms of respiratory system, but emerging evidences as mentioned above suggest that SARS-CoV-2 affects various other systems in humans as well. Clinical manifestations of multisystem infection are more unpredictable to thus make the treatment of COVID-19 more difficult. Therefore, it is necessary to perform a comprehensive physical assessment and provide a systematic therapeutic schedule for each inpatient, maybe with different symptoms. The review provides a novel perspective on COVID-19 from the infection with multisystem involvement to help the health and medical community to acquire available information. Additionally, scientific researches still need to be funded and executed to reveal more details about the molecular mechanism of SARS-CoV-2 to solve the following three solemn problems: (1) How to diagnose accurately as early as possible? (2) How to effectively control the spread of the virus? (3) How to cure the COVID-19 efficiently?

## Figures and Tables

**Table 1:** Therapeutic strategies for COVID-19.

Treatment strategy	Agents	Therapeutic target	Function mechanism
Plasma Therapy	Convalescent plasma from COVID-19 patients	Viral proteins	Transferring antibodies to neutralize the virus
Cytokine therapy	Tocilizumab, Sarilumab, Siltuximab, Sirukumab, Clazakizumab	IL-6, soluble and membrane bound IL-6R	Downregulating the JAK-STAT signaling pathway, inhibition of cytokine storm
	Anakinra	IL-1R	Inhibition of inflammatory responses and cytokine storm, alleviated lung injury
	Etanercept	TNFα	
	Mavrilimumab, TJ003234, Gimsilumab, Lenzilumab	GM-CSFR, GM-CSF	Reduced inflammatory responses and alleviated lung injury
	Bevacizumab	VEGF	Relieving lung injury
	IFNs prescription	IFN-β-1b, IFN-λ	Enhanced antiviral defense
Kinase inhibitor	Fedratinib, Ruxolitinib, Baricitinib	JAK	Blocking SARS-CoV-2 trafficking, alleviating inflammatory responses and cytokine storm, improved the lung injury
	Ibrutinib, Acalabrutinib, Zanubrutinib	BTK	Blocking B cell proliferation and cytokine release
	Sunitinib	RTK	Blocking membrane trafficking of SARS-CoV-2
	Erlotinib	EGFR	Blocking membrane trafficking of SARS-CoV-2
Cell-based therapy	NK cells transplantation	NK cells	Restoration of NK cells numbers and activity, enhanced antiviral defense
	MSC transplantation	MSC	Increased lymphocyte and anti-inflammatory cytokines and decreased pro-inflammatory cytokines, improved the lung injury
	Tregs adoption	Treg	Anti-inflammation
Monoclonal antibody therapy	Bamlanivimab	SARS-CoV-2 Spike protein	Neutralizing the virus and preventing the virus from entering the cell to proliferate
	Etesevimab		
	REGEN-COV		
	Sotrovimab		
	4A8		
Complement inhibition	Eculizumab	C5	Reduced inflammatory responses, reduced neutrophil counts, and improved lung function and lymphocyte recovery
	AMY-101	C3	
Blood purification	Cytosorb	Cytokines, DAMPs, PAMPs	Prevention of cytokine storm
